# Influence of Endurance Training During Childhood on Total Hemoglobin Mass

**DOI:** 10.3389/fphys.2018.00251

**Published:** 2018-03-21

**Authors:** Nicole Prommer, Nadine Wachsmuth, Ina Thieme, Christian Wachsmuth, Erica M. Mancera-Soto, Andreas Hohmann, Walter F. J. Schmidt

**Affiliations:** ^1^Department of Sports Medicine/Sports Physiology, Sports Science, University of Bayreuth, Universitaetsstrasse, Bayreuth, Germany; ^2^Department of Physiology, Biological Sciences, Universidad Nacional de Colombia, Bogota, Colombia; ^3^Department of Training Sciences, Sports Science, University of Bayreuth, Universitaetsstrasse, Bayreuth, Germany

**Keywords:** total hemoglobin mass, blood volume, endurance training, childhood, lean body mass, soccer, talent

## Abstract

Elite endurance athletes are characterized by markedly increased hemoglobin mass (Hbmass). It has been hypothesized that this adaptation may occur as a response to training at a very young age. Therefore, the aim of this study was to monitor changes in Hbmass in children aged 8–14 years following systematic endurance training. In the first study, Hbmass, VO2max, and lean body mass (LBM) were measured in 17 endurance-trained children (13 boys and 4 girls; aged 9.7 ± 1.3 years; training history 1.5±1.8 years; training volume 3.5 ± 1.6 h) twice a year for up to 3.5 years. The same parameters were measured once in a control group of 18 age-matched untrained children. Hbmass and blood volume (BV) were measured using the optimized CO-rebreathing technique, VO2max by an incremental test on a treadmill, and LBM by skin-fold measurements. In the second pilot study, the same parameters were measured in 9 young soccer athletes (aged 7.8 ± 0.2 years), and results were assessed in relation to soccer performance 2.5 years later. The increase in mean Hbmass during the period of study was 50% which was closely related to changes in LBM (*r* = 0.959). A significant impact of endurance training on Hbmass was observed in athletes exercising more than 4 h/week [+25.4 g compared to the group with low training volume (<2 h/week)]. The greatest effects were related to LBM (11.4 g·kg^−1^ LBM) and overlapped with the effects of age. A strong relationship was present between absolute Hbmass and VO2max (*r* = 0.939), showing that an increase of 1 g hemoglobin increases VO2max by 3.6 ml·min^−1^. Study 2 showed a positive correlation between Hbmass and soccer performance 2.5 years later at age 10.3 ± 0.3 years (*r* = 0.627, *p* = 0.035). In conclusion, children with a weekly training volume of more than 4 h show a 7% higher Hbmass than untrained children. Although this training effect is significant and independent of changes in LBM, the major factor driving the increase in Hbmass is still LBM.

## Introduction

Oxygen transport to muscles involves complex regulation that depends on hemoglobin concentration ([Hb]) and muscle perfusion. Muscle blood flow can be modulated by systemic or local regulation of vascular diameter, as well as by changes in cardiac output; for review, see Montero et al. (2015) and Mortensen et al. (2005). An important factor for a high cardiac output is a high end diastolic volume in association with a high stroke volume. Furthermore, an efficient muscular pump and fast diastolic filling are prerequisites that are only possible with an adequately high blood volume (BV). In this context, hemoglobin mass (Hbmass) is important in two ways. First, its total mass in combination with the total volume of blood determines [Hb] and O_2_ transport capacity. Second, it increases BV via an increase in erythrocyte volume (EV).

During incremental exercise, therefore, Hbmass is important and is one of the major limiting factors of maximum endurance performance. In various studies, a strong correlation between VO2max and Hbmass has been shown at all ages (Astrand, 1952; Schmidt and Prommer, 2010). It has been calculated that a change in Hbmass of 1 g results in a change in VO2max of ~4 ml·min^−1^ (Schmidt and Prommer, 2010). Therefore, endurance athletes aim to have a high Hbmass either via different training regimens (e.g., altitude training; Wachsmuth et al., 2013) or via prohibited methods (e.g., rhEPO-administration or autologous blood doping; Sottas et al., 2011).

Whether training at sea-level is also an effective way to increase Hbmass is still not entirely clear. Previous studies have found that Hbmass is unaltered in adult elite or highly trained athletes following 3 months (Glass et al., 1969; Gore et al., 1997) and 12 months (Prommer et al., 2008) of endurance training. In contrast, some authors showed a small (3%) increase in elite athletes during intensive training or between training and recovery periods (Garvican et al., 2010b; Eastwood et al., 2012b). In recreational athletes, our group observed a 6.4% increase in Hbmass following a 9-months marathon training program (Schmidt and Prommer, 2008). Their values (12.5 g·kg^−1^), however, were far lower than those observed in elite athletes (~15 g·kg^−1^) (Heinicke et al., 2001).

Based on available studies, one can conclude that training during adulthood (>21 years), exerts only small effects on Hbmass (Schmidt and Prommer, 2010). The question that arises is whether the high Hbmass observed in elite athletes is primarily inherited and/or possibly achievable through long-term endurance training during childhood and adolescence. In a cross-sectional study, Steiner and Wehrlin (2011) found a 14.5% higher Hbmass in endurance-trained adolescents at the age of 21 than in those at the age of 16 years. However, in longitudinal studies, no effects were reported after 12 months of training by cyclists aged 11–15 years (Eastwood et al., 2009) or following 18 months of endurance training in elite athletes aged 15–17 years (Ulrich et al., 2011). These results lead to the assumption that erythropoietic adaptation occurs at a very young age or during late adolescence or that Hbmass is genetically determined. The latter idea was supported by Martino et al. (2002), who observed naturally high values for Hbmass and BV, which are closely related to VO2max, in adults with no training history.

To provide more information on the possible effects of training on erythropoiesis at a very young age, the present study screened Hbmass in children at prepubertal and pubertal ages (8–14 years) following systematic endurance training over several years (>2 years) and compares the results with those from an age-matched control group. Because Hbmass is closely related to lean body mass (LBM) in adolescence, the second aim was to evaluate how growth-related increases in LBM during maturation are related to increases in Hbmass.

As the third aim, in a separate cohort, the relationship between Hbmass in prepubertal boys (7.8 years) and competition success after 2.5 years of systematic soccer training was investigated in a pilot study. As soccer performance depends to a great extent on the aerobic component of exercise, soccer players need to possess a high VO2max (Manna et al., 2010), which again is to a great extent determined by Hbmass. As the correlation between changes in VO2max and changes in Hbmass after training is low in adults (Eastwood et al., 2012a), it is assumed that Hbmass is mostly genetically determined (Martino et al., 2002) and that it might be used for talent identification not only for specific endurance disciplines, e.g., cycling (Eastwood et al., 2009), but also for team sports played on large fields like soccer.

## Materials and methods

### Participants

In the first study, 17 endurance-trained children (13 boys and 4 girls) participated in a longitudinal screening for Hbmass and endurance performance. All of them were part of a cross-country skiing training group and regularly participated in competitions. The mean training time per week was 3.5 ± 1.6 h (not including school sports) over the entire study period, and the mean training experience at the beginning of the study was 1.5 ± 1.8 years. During summer, the training included running or biking instead of cross-country skiing. Some of the participants were also part of a soccer or handball team. At the beginning of the study, the youngest participant was 7.6 years and the oldest was 11.5 years, resulting in a mean age of 9.7 ± 1.3 years (see Table 1). The control group was composed of 18 children (6 boys and 12 girls) with no experience in endurance sports. Apart from school sports (once per week), some of the subjects engaged in non-endurance sports such as equestrian, gymnastics or combat sports on a non-performance-oriented level. None of the controls had a history of endurance sports. The mean age of the controls was 11.0 ± 1.8 years. As we did not find any significant differences in anthropometric, hematological and performance parameters between male and female control subjects we established one control and one training group and did not differentiate subjects into male and female subgroups. For the anthropometric data of both groups, see Table 1. In the second study, 9 male soccer players (mean age 7.8 ± 0.2 years) underwent initial measurements of BV and Hbmass and a longitudinal screening of their performance in soccer competitions. All of the children and their parents signed a written informed consent form prior to participation. Both studies were approved by the local ethics committee at the University of Erlangen-Nuremberg, Germany.

**Table 1:** Anthropometric data (study 1).

	**Age (years)**	**Height (cm)**	**Body mass (kg)**	**BMI (kg·m^−2^)**	**Body surface (m^2^)**	**LBM (kg)**
**TRAINED (*n* = 17)**						
Initial values	9.7 ± 1.3	144 ± 11	35.9 ± 9.5	17.0 ± 2.2	1.19 ± 0.20	29.6 ± 6.4
Final values	12.4 ± 1.5^***^	158 ± 14^***^	49.5 ± 14.9^***^	19.3 ± 3.0^***^	1.47 ± 0.28^**^	39.8 ± 10.9^***^
Mean values	11.1 ± 1.3	151 ± 12	42.7 ± 11.9	18.3 ± 2.7	1.33 ± 0.23	34.9 ± 8.8
Controls (*n* = 18)	11.0 ± 1.8	147 ± 15	42.8 ± 11.2	19.4 ± 2.9	1.32 ± 0.23	32.8 ± 7.9

BMI, body mass index; LBM, lean body mass. Values are means ± SD. The term “Mean values” indicates the mean of the individual mean values of the trained subjects obtained during the whole monitoring period. Significance of differences within the trained group between initial and final values:

** *p < 0.01*,

*** *p < 0.001*.

In the following tables significance of differences are indicated as follows: Within the trained group between initial and final values: ^*^p < 0.05, ^**^p < 0.01, ^***^p < 0.001. Between the trained group and the control group: ^+^p < 0.05, ^++^p < 0.01, ^+++^p < 0.001.

### Design of the study

In the first study, the endurance-trained children were monitored for up to 3.5 years (mean observation period 2.5 ± 1.0 years), with two visits (November and April) per year, which allowed for 97 tests in total. Six of the 17 athletes (all boys) completed the study after 3.5 years and performed all 8 tests, whereas the mean number of tests per subject was 5.8 ± 2.1. At each visit, Hbmass, [Hb], hematocrit (Hct), BV and its components (plasma volume (PV) and EV), maximal endurance performance (VO2max), body mass, body height and LBM were measured. The Hbmass and BV measurements were performed 60 min after the performance test to avoid reductions in maximal performance due to blocking the oxygen binding sites by carbon monoxide (CO). For the control group, the same parameters were measured at only one visit.

In the second study, young soccer athletes were tested for BV, Hbmass, [Hb], body mass, body height, and LBM. After 2.5 years of systematic soccer training, competition performance was controlled for and quantified over 5 hierarchical success levels. Competition success was monitored over the full 2.5 years and finally ranked using a scale with the following range: (1) club level, (2) local level, (3) district level, (4) county level, and (5) up to province level.

### Procedures and protocols

In both studies, body mass was measured on a mechanical column scale while wearing shorts, a t-shirt and no shoes. Body surface area was calculated according to the formula by Mosteller (1987). Skinfold measurements (Slimguide® Caliper, Creative Health Products, Plymouth, Michigan, USA) were performed in triplicate at four sites (triceps, biceps, suprailiac, and subscapular). Typical error (TE) was 1.4%. Body fat percentages were calculated using the age-adjusted formulas of Deurenberg et al. (1990). LBM was calculated as the difference between the body mass and amount of body fat.

BVs and Hbmass were measured according to the optimized CO-rebreathing method as described by Gore et al. (2006), Prommer and Schmidt (2007) and Schmidt and Prommer (2005). The CO-bolus was adjusted to a dose of 0.6 ml·kg^−1^ for all children instead of 0.8–1.2 ml·kg^−1^ as used for adults (Prommer and Schmidt, 2007; Garvican et al., 2010a; Alexander et al., 2011). Additionally, filling of the rebreathing bag with pure oxygen was reduced to ~2 L. After familiarization with the equipment (SpiCO, Blood tec GmbH, Bayreuth, Germany) and the breathing procedure, the children performed the tests without any problems. Leakage, especially around the mouth and nose, was controlled for with a CO detector (Dräger PAC 7000, Dräger Safety, Lübeck, Germany). The typical error (TE) of this method obtained in our laboratory is 1.4%, which is in accordance with the TE published by Gore et al. (2006).

Arterialized blood samples were taken from a hyperemized earlobe to determine the [Hb] and Hct in duplicate. [Hb] was assessed photometrically (HemoCue® HB201+, HemoCue AB, Sweden), and Hct was measured by microhematocrit tube centrifugation (EBA 21, Hettich, Tuttlingen, Germany).

In addition to the blood analysis, an incremental step test to determine VO2max was performed on a treadmill (Ergo XELG2, Woodway, USA) in study 1. The initial speed was set to 6 km·h^−1^ for 3 min and increased to 8 km·h^−1^ for another 3 min. After completing the initial stages, the speed was subsequently increased by 1 km·h^−1^ each min until the subject was exhausted. Measurement of VO2max was performed using a MetaMax II device (Cortex, Leipzig, Germany), which is a portable breath-by-breath indirect spirometry system. The main criterion for the assessment of VO2max was the occurrence of a leveling-off of VO2. At this point, the VO2 values were averaged over a minimum period of 30 s to calculate the VO2max. All the children were familiarized with the treadmill before starting the test. Due to technical reasons (inaccuracy of the spirometry's O2 sensor), performance testing could not be conducted on two girls on the final date.

At each visit, the children from study 1 completed a questionnaire asking about their training volume and frequency, injuries, and illnesses within the previous 3 months. None of the children showed any severe injuries or illnesses that would have been a reason for exclusion. Training volume per week was classified into the following three categories. Category 1: 0–2 h; category 2: 2.1–4 h; category 3: >4 h.

### Data analyses

The sample size for study 1 was calculated according to Hopkins (2006). Based on the literature data of relative Hbmass in adolescents (Eastwood et al., 2009), the between-subject standard deviation was assumed to be 1.0 g·kg^−1^ and the difference between untrained children and the trained group to be 1.2 g·kg^−1^. For these numbers, the lowest sample size (50% intervention group/50% control group) was 12/12, which was surpassed in the present study (17/18).

To compare the data from the training group with data from the control group, the individual mean of each parameter was calculated for each of the trained subjects. Subsequently, these data were used in unpaired student's tests to determine possible differences between both groups. Additionally, paired *t*-tests were performed to compare the initial values of the training group with the final values at the end of the observation period.

An analysis of covariance (ANCOVA, mixed model) was performed to evaluate the influence of confounding factors on Hbmass, with the independent effects of sex and training volume as factors and age, LBM and training history as covariates. A special focus was put on the children below 12 years of age. To detect any dependencies between two variables, linear (e.g., Hbmass vs. LBM and VO2max vs. Hbmass) and exponential (age vs. Hbmass) regression analyses were performed.

In study 2, a linear regression analysis was performed to detect any relationship between Hbmass and soccer competition performance 2.5 years later.

## Results

### Anthropometric parameters

Changes in the anthropometric parameters of the training group during the period of study 1 are shown in Table 1. Body mass increased by ~5.4 kg per year, which was accompanied by an increase in LBM of 4.1 kg per year. The increase in body height was 5.7 cm per year. The controls were matched according to the mean age of the trained children at all visits, and controls did not show any significant anthropometric differences from the mean values of the training group (Table 1).

### Training data

In study 1, the children trained for more than 2 h per week for at least 3 months prior to the tests at 54% of all laboratory visits. For 29%, they had trained for more than 4 h per week, while for 17% of the visits, less than 1 h of exercise per week had been performed due to injury or illness. No endurance training was performed by the controls.

### Hematological data (study 1, all subjects)

**Figure 1:**
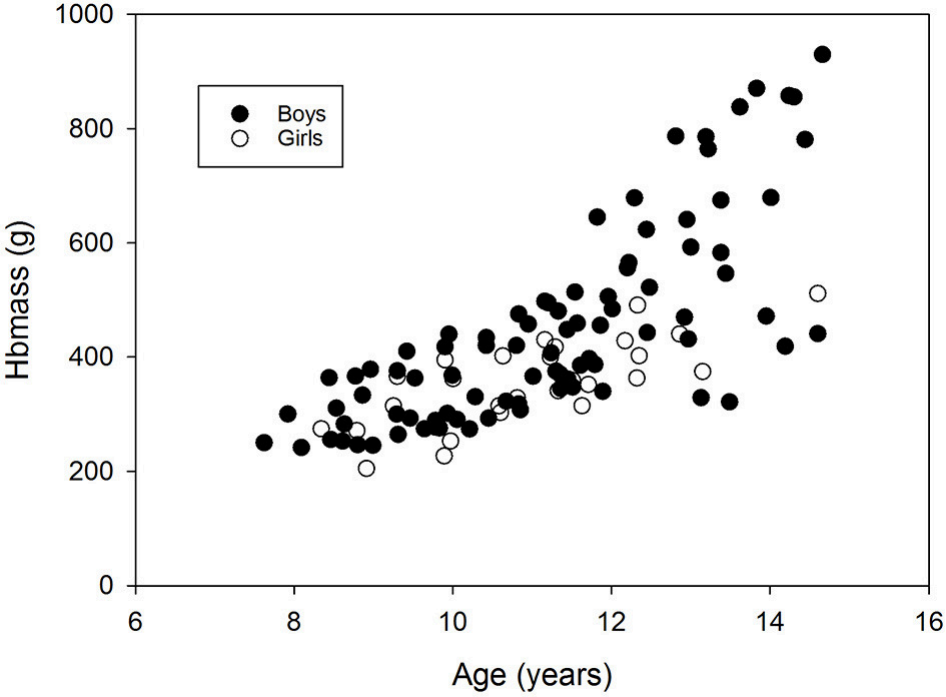
Changes in Hbmass with age. Data are from the longitudinal part of study 1 (trained children) and the cross-sectional sub-study (control group).

During the longitudinal part of study 1 (mean of 2.5 years), absolute Hbmass clearly increased by 50%. Hbmass relative to LBM showed a slight increase of 9%. While the mean absolute Hbmass did not differ from the values of the control group, relative Hbmass was elevated in the training group (Table 2). Figure 1 shows the individual Hbmass data relative to the age of the children. Despite broadly scattered results, ~200 g, there was a uniform increase until the age of 12 years. Beyond this age, no further changes were observed in the girls, although a steep increase, with a much broader spread of up to 600 g, was observed in the boys.

**Table 2:** Hematological data (study 1).

	**Hbmass (g)**	**Rel. Hbmass (g·kg^−1^)**	**Rel. Hbmass (g·kg LBM^−1^)**	**BV (ml)**	**Rel. BV (ml·kg^−1^)**	**PV (ml)**	**Rel. PV (ml·kg^−1^)**	**[Hb] (g·dl^−1^)**	**Hct (%)**
**TRAINEED (*n* = 17)**									
Initial values	338 ± 74	9.6 ± 1.2	11.5 ± 1.2	2737 ± 570	77.7 ± 8.8	1678 ± 367	47.7 ± 6.5	13.6 ± 1.1	42.5 ± 3.5
Final values	506 ± 191^***^	10.1 ± 1.3	12.5 ± 1.5^*^	3930 ± 1318^***^	79.5 ± 9.3	2391 ± 742^***^	48.7 ± 5.7	13.9 ± 0.8	42.6 ± 2.8
Mean values	420 ± 129	9.8 ± 1.0^++^	11.9 ± 0.9^+^	3364 ± 986^+^	79.1 ± 7.7^+++^	2053 ± 579	48.5 ± 5.3^+^	13.7 ± 0.6	42.7 ± 2.4^+^
Controls (*n* = 18)	370 ± 109	8.7 ± 1.1	11.2 ± 1.0	2954 ± 766	69.6 ± 7.3	1859 ± 457	44.1 ± 5.3	13.7 ± 1.0	40.4 ± 2.8

*Hbmass, hemoglobin mass; LBM, lean body mass; BV, blood volume; PV, plasma volume; [Hb], hemoglobin concentration; Hct, hematocrit. For explanation of the term “Mean values” and for statistical abbreviations, see Table 1*.

**Figure 2:**
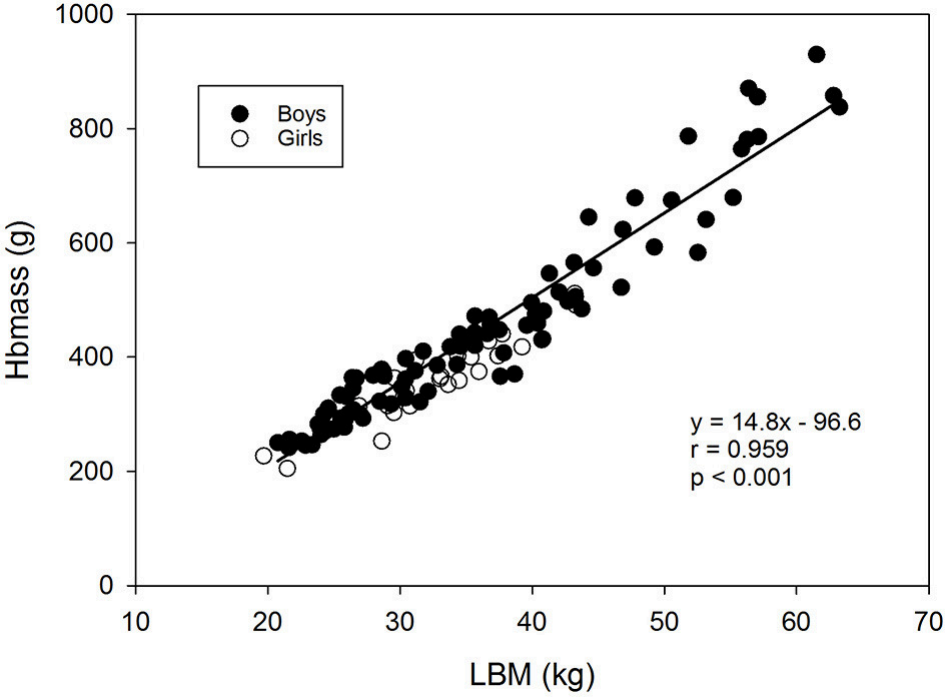
Relationship between Hbmass and lean body mass. Data are from the longitudinal part of study 1 (trained children) and the cross-sectional sub-study (control group).

Linear regression analysis with Hbmass as a dependent variable and body mass, body surface, or LBM as independent parameters, showed that the closest relationship was for Hbmass vs. LBM (*r* = 0.959, *y* = 14.8x−96.6, Figure 2) compared to Hbmass vs. body surface (*r* = 0.927) and Hbmass vs. body mass (*r* = 0.921).

Table 3 presents the results of an analysis of covariance (mixed model) for absolute Hbmass in all children below 12 years. As expected, Hbmass was primarily determined by LBM, i.e., 1 kg LBM increased Hbmass by 11.4 g, while age had no effect and sex had only a small effect (28.7 g higher in boys). Additionally, training volume exerted a small but highly significant effect, with the group with highest training volume (>4 h/week) showing a 25.4 g higher Hbmass than the group with lowest training volume (<2 h/week). Training history did not have a significant influence.

**Table 3:** Results of ANCOVA for absolute Hbmass in children below the age of 12 years (study 1).

	**Unit**	**ANCOVA *p*- value**	**Regression weights**	**95% Confidence interval**
Base		0.802		
Sex	Males	0.022	28.7	4.5; 53.0
Age	Years	0.93		
Activity level		0.001		
	2 h ≤ 4 h	0.135	11.4	−4.5; 27.3
	>4 h	0.001	25.4	11.6; 39.2
Training history	Years	0.90		
LBM	(kg)	0.000	11.4	9.0; 13.7

*LBM, lean body mass*.

Very similar results were obtained from the mixed model when all the data (i.e., also including those older than 12 years) were included. In this case, the effect of training volume on absolute Hbmass was +22.5 g (>4 h/week, *p* < 0.001), the effect of sex was 25.0 g (*p* < 0.025), and the effect of LBM was 12.4 g·kg^−1^ LBM (*p* < 0.001).

The age-related increase in absolute PV and BV mirrored the time course of changes in Hbmass (Table 2), showing an increase in PV by ~700 ml and in BV by ~1,200 ml. The volumes in relation to body mass remained constant over the observation period. Additionally, [Hb] and Hct did not change over time (Table 2).

### Hematological data from the male training group (study 1, longitudinal data)

**Figure 3:**
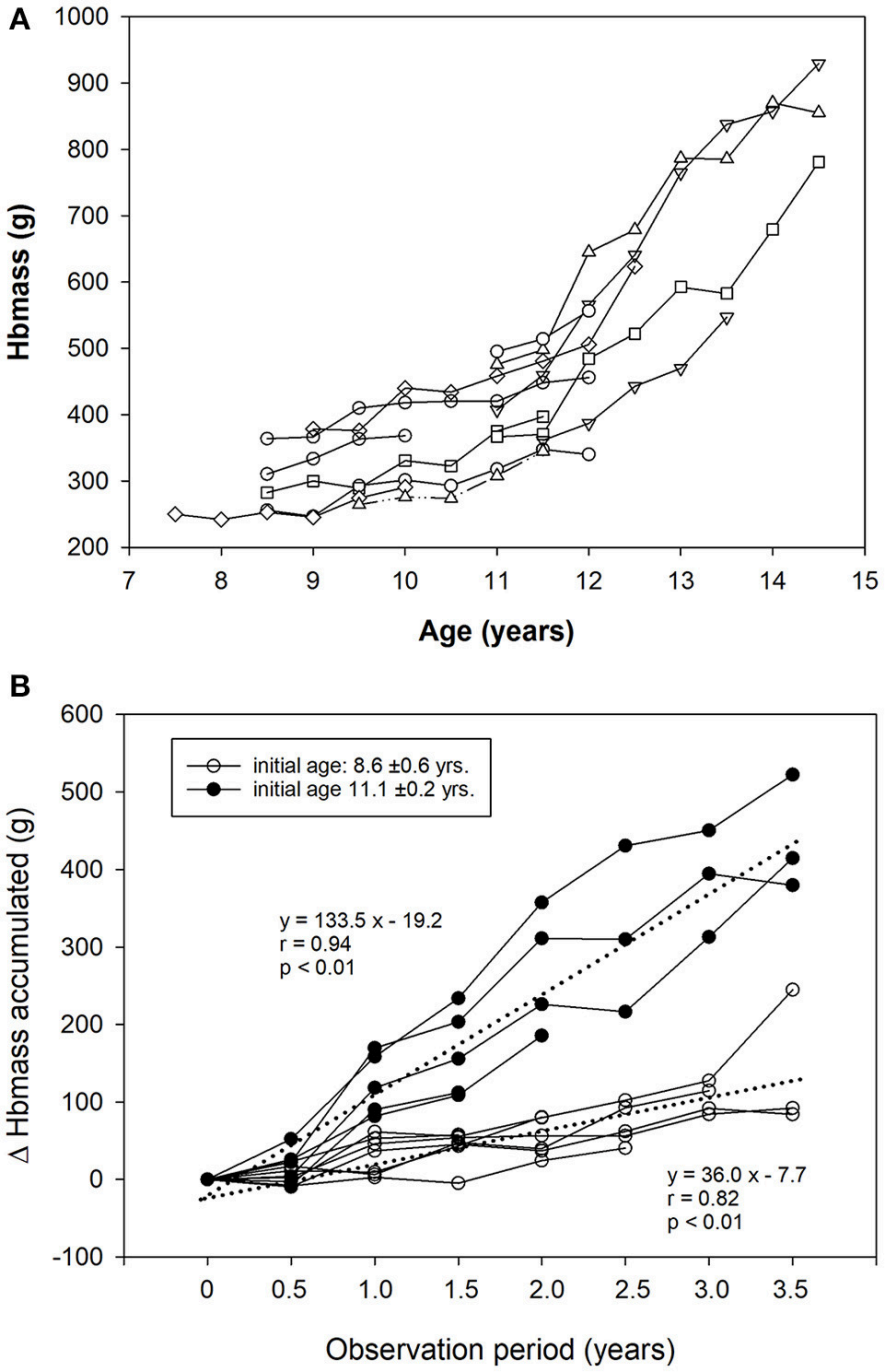
**(A)** Changes in Hbmass with age in trained boys (longitudinal part of study 1). **(B)** Accumulated increase in Hbmass over the 3.5-year observation period (trained boys only). The dashed lines indicate the regression lines for data from children who entered the study at the age of 8.6 and 11.1 years.

The changes in the boys' Hbmass are separately highlighted in Figure 3. We found a linear increase in Hbmass until the age of 12 years and an almost exponential increase thereafter (*r* = 0.87, *p* < 0.001, Figure 3A). Boys who started the study at a mean age of 8.6 ± 0.6 years increased their Hbmass by 36 g per year, while those who started at 11.1 ± 0.2 years increased their Hbmass by 133 g·yr^−1^ in the following 3.5 years. (Figure 3B).

### Performance data (study 1)

Absolute VO2max increased in the male group by almost 400 ml·min^−1^ but slightly decreased when compared to body mass (Table 4). Absolute and relative VO2max were moderately elevated in the training group (*p* < 0.05) compared to values in the control group. The maximum speed attained on the treadmill slightly increased over time and was significantly higher in the training group (Table 4).

**Table 4:** Performance data (study 1).

	**Abs. VO_2max_ (ml·min^−1^)**	**Rel. VO_2max_ (ml·kg^−1^·min^−1^)**	**Max. speed (km·h^−1^)**	**Max. heart rate (b·min^−1^)**	**RER**
**TRAINED (*n* = 17)**					
Initial values	1,969 ± 448	56.0 ± 6.4	15.0 ± 1.7	204 ± 10	1.23 ± 0.08
Final values	2,382 ± 6^***^	50.1 ± 5.1^**^	15.6 ± 1.6^*^	203 ± 11	1.25 ± 0.10
Mean values	2,297 ± 612^+^	54.4 ± 5.2^+++^	15.4 ± 1.5^+++^	203 ± 10	1.23 ± 0.07
Controls (*n* = 18)	1,898 ± 475	44.9 ± 5.6	12.1 ± 1.7	198 ± 6	1.18 ± 0.10

*RER, respiratory exchange ratio. For explanations of the term “Mean values” and for statistical abbreviations, see Table 1*.

### Anthropometric and hematological data from the group of soccer players (study 2)

Table 5 presents the anthropometric and hematological results from the child soccer players in study 2 at the beginning of the study. We found normal BV and Hbmass levels at the age of 7.8 years. When the hematological data were related to soccer performance after a period of 2.5 years, we found a significant relationship between Hbmass (g·kg^−1^) and performance (*r* = 0.627, *p* = 0.035).

**Table 5:** Anthropometric and hematological data of the participants of study 2.

	**Age (years)**	**Height (cm)**	**Body mass (kg)**	**LBM (kg)**	**Hbmass (g)**	**Rel. Hbmass (g·kg^−1^)**	**Rel. Hbmass (g·kg LBM^−1^)**	**BV(ml)**	**Rel. BV (ml·kg^−1^)**	**Rel. BV (ml·kg LBM^−1^)**	**[Hb] (g·dl^−1^)**
Mean ± SD	7.8 ± 0.2	125 ± 5	24.5 ± 2.7	22.6 ± 2.5	250.8 ± 23.7	9.8 ± 0.8	11.3 ± 0.4	2,186 ± 206	85.0 ± 6.1	98.4 ± 3.0	12.6 ± 0.4
Minimum	7.5	119	22.0	19.9	226.8	8.3	10.9	2,017	73.9	94.1	12.0
Maximum	8.1	133	29.7	27.5	298.7	10.7	12.1	2,628	90.4	102.2	13.1

*n = 9; for further explanations, see Table 1*.

## Discussion

The aim of study 1 was to screen preadolescent children (9.7 ± 1.3 years) for a period of 3.5 years to investigate the influence of systematic endurance training on Hbmass. While comparable studies have investigated older adolescents or shorter intervention times (von Dobeln and Eriksson, 1972; Eastwood et al., 2009; Steiner and Wehrlin, 2011; Ulrich et al., 2011), this study shows that Hbmass might be influenced at a very young age by long-term endurance training. The main finding from the present study, however, is that the increase of Hbmass over time is mostly driven by changes in LBM, which overlaps with the effect of age but is additionally influenced by the sex of the individual and training volume.

Furthermore, study 2 showed that an early high Hbmass might already have a positive influence on later competition success in youth soccer for children between the ages of 7.8 ± 0.2 years and 10.3 ± 0.3 years.

### Development of Hbmass during adolescence

In study 1, the mean values for Hbmass of the athletes (~420 g) and controls (~370 g) was similar to published data, showing a mean value of ~430 g in 11–13-year-old boys (von Dobeln and Eriksson, 1972) and ~370 g in 12–13-year-old girls and boys (Astrand, 1952).

Below the age of 12 years, Hbmass was slightly higher in boys than in girls (estimate by ANCOVA was ~25 g), which agrees with the data of Karlberg and Lind (1955). Hbmass showed a very similar increase in both sexes until the onset of puberty (Figure 1, ~36 g·yr^−1^). Afterwards, the increase became considerably less in girls, while the increase was almost exponential in boys (133 g·yr^−1^).

Despite the obvious increase in Hbmass with age, ANCOVA did not show any independent statistical effects of age on Hbmass, as the effects fully overlapped with those of LBM. As demonstrated in Figure 2, linear regression analysis showed a strong relationship (*r* = 0.959, *p* < 0.001) between both parameters, demonstrating that a 1 kg increase in LBM was associated with a 14.8 g increase in Hbmass. This close relationship has been shown for adults in various recent studies (Sawka et al., 1992; Schumacher et al., 2008) but has not previously been shown for children, and the present results prove that the development of LBM, to a great extent, explains changes in Hbmass. A very similar picture as that for Hbmass was found for BV with respect to the percentage change, time course and statistical dependency on LBM, which is in agreement with previous studies (Astrand, 1952; Karlberg and Lind, 1955; von Dobeln and Eriksson, 1972). We, therefore, conclude that LBM is a strong predictor of Hbmass and BV in children, as this parameter indirectly incorporates age, height, and body mass.

When analyzing the development of Hbmass as a function of age, it is apparent that Hbmass increases exponentially at the age of ~12 years in most boys (Figure 3A). Whether this sharp increase reflects puberty-associated changes in erythropoiesis can only be speculated as more specific data on maturation were unfortunately not obtained in this study. However, data from the literature showing a close relationship between the increase in testosterone levels during puberty and [Hb] (Krabbe et al., 1978; Thomsen et al., 1986; Hero et al., 2005) support our hypothesis that Hbmass is affected by androgens and that the increase in Hbmass in the boys starting at the age of ~12 years is directly due to increasing testosterone levels.

In females, the lower level of testosterone relative to that in male adolescents is probably the reason for the plateau in Hbmass, which becomes obvious during and after puberty (Astrand, 1952; Tanner, 1962). We consider the lack of capacity for further increases in Hbmass as one reason for the early peak in performance in women, which is frequently observed by the age of 14 years in, for example, swimming (Kojima et al., 2012).

### Influence of endurance training on Hbmass

It is a well-known fact that elite endurance athletes possess an ~40% higher Hbmass than sedentary subjects (Heinicke et al., 2001); in top individual athletes, differences in Hbmass greater than 70% have been observed (Heinicke et al., 2001). One important reason is most likely a training-independent genetic predisposition, as subjects with high rel. VO2max (65 ml·kg^−1^·min^−1^) but without any training history are characterized by a 24% higher Hbmass and 16% higher BV than subjects with low rel. VO2max (46 ml·kg^−1^·min^−1^) (Martino et al., 2002). Therefore, a strong genetic impact can be assumed.

The impact of the training itself on accelerated erythropoiesis has been discussed and the findings are controversial. In adult elite endurance athletes, changes in training volume and intensity showed no (Prommer et al., 2008) or only very small effects (3%, Garvican et al., 2010b) on Hbmass. Higher effects (6.4%) have only been demonstrated in recreational athletes preparing for a marathon competition over 9 months (Schmidt and Prommer, 2008). Thus, the available data demonstrates that training during adulthood only yields negligible stimulating effects on Hbmass, which cannot explain the large differences between endurance athletes and sedentary subjects. Therefore, the hypothesis arises that training during adolescence or even childhood may have an essential impact on erythropoiesis. This idea is also supported by the fact that East African runners start with high-volume training at very early stages in life (Prommer et al., 2010).

Today, there are four studies available highlighting this controversial point. Steiner and Wehrlin (2011) compared Hbmass values of elite endurance athletes at 16, 21, and 28 years and reported 15% higher values for the 21-year-old athletes than for the 16-year-old athletes. However, no further increases were observed from 21 until 28 years of age. They concluded that the period between 16 and 21 years is a very sensitive phase for training effects, whereas training at a younger age appeared to have negligible effects with respect to enhanced erythropoiesis. Ulrich et al. (2011) monitored the Hbmass of 15–17-year-old boys and girls during a 1.5-year training period. They found a 15% higher Hbmass in the trained subjects than in the untrained subjects but found no training effects. Very similar results were demonstrated for 11–15-year-old boys and girls; these results showed 10% higher values in the athletic group (cyclists) than in the sedentary subjects. Changes in Hbmass occurring during the one-year training period were attributed to the normal maturation process and not to the training itself (Eastwood et al., 2009). In the only study conducted with younger children (mean age 11.8 years) (von Dobeln and Eriksson, 1972), an increase in Hbmass of 39 g after a 16-week training program was attributed solely to normal growth in the children. These results coincide well with the increase of 133 g·yr^−1^ observed in this study for this age group (Figure 3B), corresponding to a change of 41 g/16 weeks. In the present study, which first monitored Hbmass in children between 8 and 12 years and then monitored it over a longer training period of up to 3.5 years, a training effect of approx. 25 g (corresponding to 7%) was demonstrated for Hbmass (Table 3). This effect was not statistically related to the effect of LBM, which suggests the existence of an independent training effect not due to normal growth mechanisms. However, as we did not include a longitudinal control group during the training period, we cannot determine whether the 7% increase related to training is due to a direct training effect or due to a selection process that favors children with a naturally high Hbmass (Eastwood et al., 2009; Ulrich et al., 2011). In any case, the 7% higher values related to training cannot explain the large differences between trained and untrained subjects in adulthood. As was shown for VO2max (Bouchard et al., 1998, 1999; Martino et al., 2002), we hypothesize a high basic genetic impact and a genetically determined influence of endurance training on Hbmass, which, according to Steiner and Wehrlin (2011), may preferentially occur in late puberty.

### Aerobic performance

VO2max in our athletic group was 21% higher than in the control group and 24% higher than in a group of untrained children between 11 and 13 years (von Dobeln and Eriksson, 1972). During the monitoring time, absolute VO2max increased by ~400 ml, which is in line with a meta-analysis including data from 2100 boys between 6 and 16 years (Bar-Or and Rowland, 2004). In contrast, relative VO2 max decreased by 5.9 ml·kg^−1^·min^−1^, which is normally observed in girls above the age of 11 years but not in boys. One reason may be the relatively high baseline values obtained during the period when all participants performed intensive endurance training; however, with increasing age, the training volume was reduced in some of the boys.

**Figure 4:**
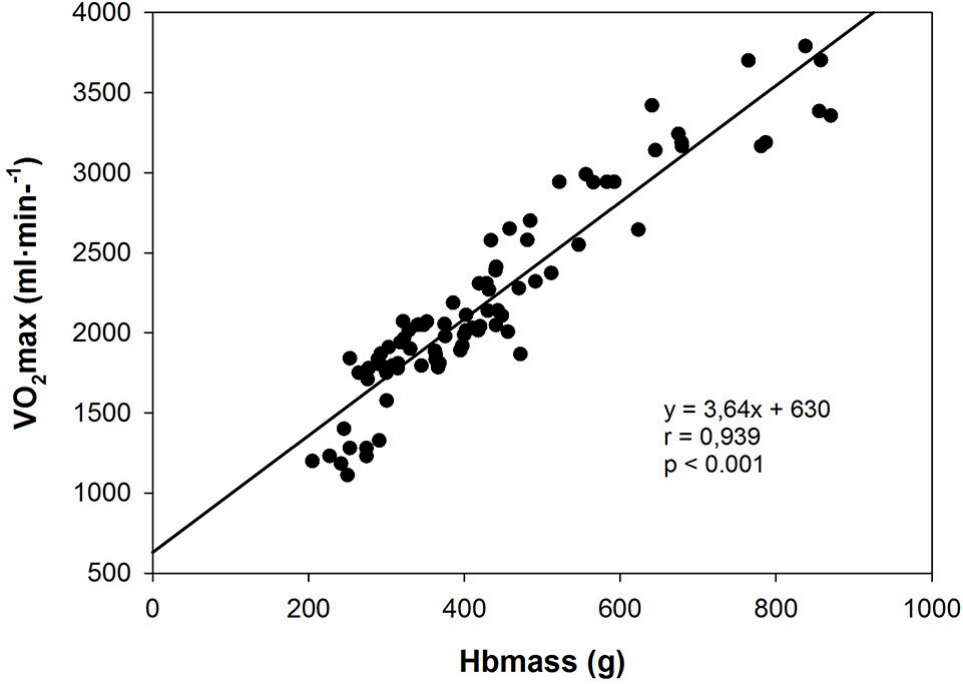
Relationship between VO2max and Hbmass. Data are from the longitudinal part of study 1 (trained children) and the cross-sectional sub-study (control group).

The strong dependency of VO2max on Hbmass (*r* = 0.939, Figure 4) is demonstrated by the slope of the regression line (3.64); this indicates that a 1 g change in Hbmass results in an ~4 ml·min^−1^ change in VO2max. As nearly identical slopes were found in several studies with adults (Gore et al., 1997; Schmidt and Prommer, 2010), we can conclude that Hbmass exerts the same impact on the oxygen transport system in children as in trained and untrained adults. During incremental exercise, therefore, Hbmass gains importance as a limiting factor, in combination with a high level of muscle perfusion and a high cardiac output, in the maximum supply of oxygen to working tissues.

### Prognostic relevance of Hbmass

The significant relationship (*r* = 0.627, *p* = 0.035) in our pilot study on the prognostic relevance of blood parameters, in this case between Hbmass and performance level in soccer competitions 2.5 years later in prepubertal boys (7.8 years), hints to a possible relevance of the aerobic component of exercise for soccer performance. This is in line with Eastwood's et al. hypothesis of the prognostic validity of Hbmass for talent identification (Eastwood et al., 2012a) not only in pure endurance disciplines but also in soccer, where the long distances covered during a game make high demands on the endurance of the players.

### Study limitations

The main limitation of both studies is the small number of athletic children, especially of girls in study 1. A lack of a control group in study 1 that was monitored over the same time period and with the same frequency as the athletic group makes it difficult to distinguish between real training effects and genetic predispositions. Furthermore, a mean training volume of 3.5 h/week may be too low to achieve relevant increases in Hbmass. In contrast, it is very difficult to monitor young children with regard to a continuous and high training impact over several years as children do not focus on high-performance training groups at this age and the drop-out rate is quite high. Therefore, the data presented here provide valuable insights into changes in Hbmass under a relatively intensive training burden in prepubertal children.

## Conclusion

Hbmass linearly increases between the ages of 8 and 12 years in both sexes, showing a mean increase of ~25 g·yr^−1^. Beyond 12 years, increases in Hbmass are almost exponential in boys, with a change of ~130 g·yr^−1^ probably reflecting the impact of testosterone production during puberty. These age-related changes in Hbmass are mainly promoted by the development of LBM, although long-term endurance training (>4 h/week) exerts additional effects (~7%). However, this study cannot definitively conclude whether the higher Hbmass found in endurance-trained children below the age of 12 years is due to the training itself or due to genetic preselection. Also, some limitations of this study, such as the low number of female athletes and the lack of collection of cross sectional data in the control group, require a cautious interpretation of our results. However, our data hint at positive effects of a high Hbmass at an early age on subsequent competition performance in, for example, soccer.

## Author contributions

NP, IT, AH, and WS: conceived the study; NP, NW, IT, EM-S, CW, AH, and WS: contributed to data collection, with NP and WS performing all statistical analyses; NP, AH, and WS: drafted the manuscript, to which NW, IT, EM-S, and CW then contributed. All authors read and approved the final version of the manuscript; NP, NW, IT, EM-S, CW, AH, and WS: agree to be accountable for all aspects of the work in ensuring that questions related to the accuracy or integrity of any part of the work are appropriately investigated and resolved.

### Conflict of interest statement

WS and NP are managing partners of the company “Blood tec GmbH,” who provided the required equipment and expertise during this study for the measurement of hemoglobin mass using the optimized carbon monoxide rebreathing method. NW, IT, EM-S, CW, and AH do not have any potential conflict of interest.
